# Relevance and flexibility are key: exploring healthcare managers’ views and experiences of a de-adoption programme in the English National Health Service

**DOI:** 10.1186/s12913-025-12700-1

**Published:** 2025-04-24

**Authors:** Nicola Farrar, Carmel Conefrey, Mike Bell, Jane Blazeby, Christopher Burton, Jenny Donovan, Andy Gibson, Joel Glynn, Tim Jones, Josie Morley, Angus McNair, Amanda Owen-Smith, Ellen Rule, Gail Thornton, Victoria Tucker, Iestyn Williams, William Hollingworth, Leila Rooshenas

**Affiliations:** 1 Population Health Sciences, Bristol Medical School, University of Bristol, Canynge Hall, 39 Whatley Road, BS8 2PS Bristol, UK; 2 National Institute for Health and Care Research Applied Research Collaboration West (NIHR ARC West) at University Hospitals Bristol and Weston NHS Foundation Trust, Bristol, UK; 3 Bristol Centre for Surgical Research, Bristol Medical School, Population Health Sciences, University of Bristol, Bristol, UK; 4 NIHR Bristol Biomedical Research Centre, University Hospitals Bristol and Weston NHS Foundation Trust and University of Bristol, Bristol, UK; 5 School of Allied and Public Health Professions, Canterbury Christ Church University, Canterbury, UK; 6 Musculoskeletal Research Unit, Translational Health Sciences, Bristol Medical School, Southmead Hospital, Bristol, UK; 7 North Bristol NHS Trust, Bristol, UK; 8 Gloucestershire Integrated Care Board (ICB), Brockworth, UK; 9 Bristol, North Somerset, and South Gloucestershire Integrated Care Board (ICB), Bristol, UK; 10 Health Services Management Centre, University of Birmingham, Birmingham, UK

**Keywords:** Qualitative, Healthcare commissioning

## Abstract

**Background:**

De-adoption of healthcare involves stopping or removing provision of an intervention, usually because of concerns about harm, effectiveness, and/or cost-effectiveness. De-adoption is integral to upholding the quality and sustainability of healthcare systems, but can be challenging to achieve. Previous research conducted with healthcare decision-makers identified a desire for more national support to identify and implement de-adoption opportunities. The ‘Evidence-Based Interventions’ (EBI) programme was a de-adoption programme introduced in the English National Health Service (NHS), comprising national recommendations to guide provision of over 40 healthcare interventions. This study aimed to investigate commissioners’ actions in response to this initiative, providing insights to improve the success and impact of future de-adoption programmes.

**Methods:**

This was a qualitative study, employing in-depth, semi-structured interviews with NHS commissioners. Interviews were analysed thematically using the constant comparison approach. This work was part of a wider mixed-methods study, which aimed to investigate the delivery, impact, and acceptability of the EBI programme across the NHS.

**Results:**

Twenty-five interviews were conducted with 21 commissioners from 7 regions of England. Although commissioners were supportive of the ethos of using evidence-based criteria to guide equitable provision of care, they described inconsistent or limited adoption of EBI recommendations. Commissioners questioned the value and relevance of the recommendations, which often targeted interventions with pre-existing local policies. Local policies often set higher thresholds for accessing interventions, raising concern that adoption of national policies would raise activity to an unsustainable level given strained budgets. Interviews also revealed how implementation of national de-adoption recommendations was not a straightforward process, as they still needed to pass through multi-faceted local ratification processes, which required time, resource, and information/justification that was not always available, making implementation problematic.

**Conclusion:**

This study is, to our knowledge, the first investigation of how devolved healthcare policymakers respond to national de-adoption recommendations. Our study highlights that local implementation of national de-adoption policies is not necessarily straightforward, by virtue of the fact that de-adoption concerns entrenched interventions for which devolved policies may already exist. It is therefore critical that national de-adoption initiatives provide guidance around how devolved policymakers should reconcile national recommendations with local policies and processes.

**Supplementary Information:**

The online version contains supplementary material available at 10.1186/s12913-025-12700-1.

## Background

Introduction of a new healthcare technology into practice typically requires evidence of safety, effectiveness, and cost-effectiveness, but many interventions in current use predate these standards. As evidence evolves, there is increasing recognition of the importance of de-adopting (stopping or reducing) interventions that are not as effective or cost-effective as once presumed [1], particularly in the context of finite resources [2]. There is increasing recognition that de-adoption is critical to sustaining healthcare systems and improving care, but it is notoriously difficult to achieve [3].

Several de-adoption programmes have been implemented in healthcare systems at the meso-level (e.g. within an individual hospital) or macro-level (e.g. via a national initiative), albeit with mixed success [4, 5]. The evidence-base around strategies to de-adopt complex interventions, such as surgery, is particularly limited, with most of the evidence to date relating to medication [6]. The international ‘Choosing Wisely’ campaign initially showed limited impact in reducing the targeted healthcare practices including surgical procedures [7], and has since been shown to be more effective if used with other interventions, such as those targeting clinicians [8]. In 2019, NHS England launched its ‘Evidence-Based Interventions’ (EBI) programme, which aimed to “*reduce the number of medical or surgical interventions as well as some other tests and treatments which the evidence tells us are inappropriate for some patients in some circumstances*” (https://www.aomrc.org.uk/ebi/about/). The EBI programme’s first list of interventions, “List 1” was launched in 2019. It included 17 surgical interventions and guidance for reduction in their use. A second list of 31 interventions, tests and procedures was published in November 2020 (List 2) [9]. Clinical Commissioning Groups (CCGs) (replaced with Integrated Care Boards (ICBs) in July 2022) were expected to implement the EBI recommendations for Lists 1 and 2 interventions within their geographic area. These groups are responsible for purchasing healthcare for their local populations, in the context of the English NHS. The commissioning process includes retrospectively reimbursing hospitals for episodes of elective care based on a National Tariff. Commissioners typically develop their own policies to guide provision of treatments, tests, or procedures within their geographic region, often stipulating criteria that must be met for a patient to access them. Criteria may include requirements to try less invasive treatments prior to an intervention, thresholds for symptom severity, or diagnostic thresholds, while some policies also specify lifestyle criteria for accessing an intervention (e.g. smoking cessation or weight loss) [10, 11].

Previous research examining local/meso-level decision-makers’ approaches to de-adopting healthcare has illuminated barriers to policymaking and implementation in this area. Both Elshaug [3] and Rooshenas [2], when exploring the perspectives of local/regional health policy makers in Australia and England respectively, identified a lack of formal processes to support ‘disinvestment’, concerns about public responses to de-adoption, and a desire for more central/national support in promoting de-adoption agendas. The EBI programme, in theory, served as a solution to some of these issues. To our knowledge, there has been no research to examine how devolved healthcare purchasers respond to national de-adoption initiatives such as the EBI programme.

The aim of the research reported here was to explore actions taken by commissioners in response to the EBI programme and their perceptions of the acceptability and potential consequences of these actions. This work is part of the wider ‘OLIVIA’ study, which aims to investigate the delivery, impact and acceptability of the EBI programme across CCGs/ICBs in the English NHS, with a view to producing evidence-based recommendations to guide future de-adoption of healthcare [12].

## Methods

This qualitative study consisted of in-depth, semi-structured interviews with NHS commissioners concerning their perspectives on the delivery, impact, and implementation of the EBI programme.

### Sampling of CCGs

The project set out to understand responses to the EBI programme by focusing data collection efforts in regions that represented extremes in the extent to which they had reduced activity rates for selected procedures from List 1 of the EBI programme: tonsillectomy for recurrent tonsillitis, Dupuytren’s contracture release, and arthroscopic shoulder decompression for subacromial pain. These procedures were selected purposefully, with the intention of varying key characteristics that were anticipated to shape de-adoption success, including: existing evidence for each procedure [13–16] (e.g. whether there were any RCTs underway or published), the availability of alternative treatments (e.g. medication or physiotherapy), and the nature of the surgical speciality itself. The three selected procedures represented variability in relation to these considerations. Patient and public representatives, who were involved throughout the design and conduct of this research, contributed to their selection.

For each of the three procedures, ten CCGs that had reduced procedure rates by the most and ten that had reduced procedure rates by the least were identified to target for participant recruitment (see Supplementary material 1, for more details around how the rankings were generated).

### Recruitment of participants

The research manager at the host CCG collaborating on the OLIVIA study or the first author sent out initial email invitations to commissioning bodies, requesting interviews with staff involved in the commissioning policy development process. Non-responders were followed up at two weeks. Further information, including a copy of the study information sheet and the consent form, were sent to respondents and any questions were addressed. An MS Teams invitation was sent to respondents who indicated an interest in participation. Throughout data collection, it became increasingly apparent that the rankings in procedure rate reduction did not align with policy-related changes or commissioning practices. This was corroborated in two studies – one from the OLIVIA study and one from Anderson et al. – which showed that reductions in procedure rates were minimal on a national scale [9, 17], concluding that List 1 of the EBI programme had limited impact. It also became apparent that the issues emerging from commissioners’ interviews were not specific to any given procedure – rather, they were generic issues related to the EBI programme and de-adoption processes. As such, following the initial 10 interviews, we opted to further develop the generic emerging issues by expanding our sampling efforts. We took a snowball sampling approach [18], whereby respondents identified others within their regional or national networks who may be suitable to take part, and sought to ensure our emerging sample of informants reflected geographic spread.

### Data collection

All interviews were conducted by NF – a post-doctoral researcher with expertise in qualitative methodology, who had no previous or ongoing association with any of the participants. A verbal consent form was used and all interviews were audio-recorded with consent. A copy of the completed consent form was sent to all participants. A topic guide (Supplementary material 2) was used to guide the interviews and was revised throughout the course of data collection as new topics of relevance emerged from concurrent analysis. The topic guide asked about commissioners’ perspectives of the EBI programme in general, and initially included more focused questions on the actions taken in relation to the three procedures. The topic guide evolved throughout the data collection process, as although the original intention was to focus on List 1, timings of the interviews meant that participants often reflected on their experiences of List 2 and the consultation period for List 3, published in May 2023, as they had occurred more recently.

### Data analysis

Data were analysed thematically, using the constant comparison approach adopted from Grounded Theory [19]. The first author led the analysis of this study, taking an inductive approach. Transcripts were imported into NVivo [20] to facilitate coding. The transcripts were read and extracts were assigned codes to reflect explicit and implicit content. Codes concerning related concepts were grouped into themes and subthemes. Themes were regularly reviewed during analysis and iteratively revised as the analysis evolved. Three initial transcripts were double-coded by experienced qualitative methodologists (CC and LR – both non-clinical researchers, with expertise in qualitative methodology and health services research). Updates on developing findings were regularly discussed by NF, CC and LR, and anonymised findings were periodically presented to the wider project team (consisting of academics, clinicians, commissioners, and patient/public representatives, none of whom had any association with the research participants). A descriptive account of the findings was iteratively developed, and shared with LR, CC, WH, TJ, JG and GT for comments. Subsequent to the initial descriptive account, the team agreed that further data collection would be beneficial to gain a deeper understanding around some of the developing themes, which continued to be refined through further data collection and ongoing analysis. An ‘inductive thematic’ approach to saturation was taken [21], continuing data collection and analysis until no new themes were being drawn from the data in relation to our study objectives. Assessments of data saturation were undertaken by NF, CC, WH, and LR, whom regularly scrutinised summaries of emerging findings via review of descriptive accounts and discussion in team meetings. Negative cases (where participant responses appeared to contradict emerging analysis) were discussed in order to ensure the findings reported comprehensively considered the variation and complexity of participants’ accounts.

As data collection and analysis progressed, it became clear that many commissioners were not in a position to offer specific insights into the three procedures identified for discussion for this study, including why their CCG had seen a greater or lesser reduction in the number of procedures undertaken. Accordingly, although participants were all asked whether they could comment on the particular procedures, the topic guide evolved to encourage more general reflections on the wider impact of the EBI programme and where it ‘fit’ within local commissioning structures, resulting in a pivot away from a focus on commissioner responses to particular procedures that was initially intended.

### Context of research

The consequences of the COVID- 19 pandemic on commissioners (as well as the healthcare system) were wide reaching, impacting both workload and priorities. In addition, the cessation of CCGs as commissioning bodies and the introduction of ICBs was announced during this project and implemented in July 2022. This also impacted commissioners’ workloads, the structures in which they worked, and, in some cases, the geographical boundaries across which their work applied. The relevance of these contextual factors are discussed throughout the results and discussion.

## Results

Twenty-five interviews were conducted with 21 participants between August 2021 and September 2022, ranging from 27 to 80 min (average 46 min) in duration. Repeat interviews were conducted with four participants to further understand their perspectives and experiences of List 2. The sample included participants responsible for drafting commissioning policies, and those with commissioning management roles and clinical oversight responsibilities. As part of their roles, many participants had EBI implementation or oversight responsibilities. Approximately half of the participants were clinically trained (10/21). The locations of participants’ respective commissioning organisations were spread across England, as shown in Table 1.

**Table 1:** Geographic and training characteristics of participants

Characteristics of participants (geographical and training)		Number of interviewees	% of all participants
Geographic distribution of CCGs	London	2	10
	North East	0	0
	North West	1	5
	Yorkshire	1	5
	East Midlands	2	10
	West Midlands	5	24
	South East	4	19
	East of England	0	0
	South West	6	29
Clinical training of participants	Clinically trained (e.g., doctor, nurse, pharmacist)	10	48
	Non-clinically trained	11	52

### Overview and presentation of findings

Two overarching themes were identified that appeared to explain commissioners’ responses to the EBI de-adoption programme: the perceived need, value, and relevance of the EBI programme, and the role of local context in shaping adoption of the EBI recommendations. These two broad themes are discussed, with sub-themes, in the sections that follow, with illustrative quotes throughout. Negative cases, where present, are presented to provide a comprehensive account of participants’ responses.

### Perceived need, value, and relevance of the EBI programme

#### Support for the ethos of a national evidence-based de-adoption programme

One of the stated ambitions of the EBI programme was to reduce variation in surgical procedure rates across England, through encouraging CCGs to adopt national criteria underpinned by the latest evidence. The principle of ensuring criteria for accessing interventions were ‘evidence-based’ resonated with many commissioners, some of whom conveyed the integral role of evidence in their usual local policy development processes:

C0014: “*We [the team] used to be called ‘Individual Funding’. Our name was changed about three years ago to ‘Evidence-Based Interventions’ because the CCG felt that we were more about evidence base because we deal with policies as well*,* and when applications come through*,* we are looking at the evidence. So they decided to change the name. It was more appropriate to have Evidence-Based Interventions*.”

Throughout the interviews, commissioners often showed their support for tackling unwarranted variation when describing the benefits of the EBI programme, seeing it as something that could help to avoid differential access to interventions based on where one lives:

C008: “*I agree with EBI. I think if you just look at what they are trying to do*,* which is reduce or minimise unwarranted variation*,* well*,* yes*,* absolutely. Why should there be a postcode lottery? Why should patients in different areas be able to access [different] care? Why should that be happening differently around the country*?… *So I think the programme works*,* and I think it has allowed there to be a conversation on a national level*.”

Despite appreciation for the ethos of the EBI programme and its potential to tackle issues of importance to commissioners, all informants described inconsistent or limited implementation of the EBI programme’s de-adoption recommendations. The findings that follow explain the challenges around implementation.

#### Impact of EBI limited by overlap with pre-existing local policies

Commissioners’ accounts of the List 1 EBI recommendations were dominated by the rhetoric that these overlapped with local pre-existing policies. Similar to the national EBI recommendations, their local policies also specified criteria for accessing these procedures:

C002: *“We started trying to implement a policy about things that required prior approval*,* or things that could only be done if certain criteria were met*,* about 18 years ago.”*

Commissioners often described their CCGs’ responses to List 1 as being limited, in part because the procedures targeted were seen as being the “*usual suspects*” (C007) and broadly the types of interventions where local policies already existed:

C007: “*And to be honest - and we’ve said this to NHS England - is that the Phase One [List One] really didn’t make much difference to us. Because we pretty much had 16 out of the 17 Phase Ones in our existing policy suite.”*

The perceived overlap of EBI recommendations with existing local policies were framed by some as a missed opportunity for the national programme to tackle policy-development in areas they found contentious, such as “big volume, big money” (CO18) interventions that were deemed politically charged. Assisted conception and cataract surgery were two such examples given, for which access to care varied geographically, and policies were considered difficult to develop due to the perception of them being emotive issues:

C011: “*They [EBI] could have actually worked on something really*,* really meaty and gritty and been ground-breaking with it*,* and they actually decided to play it safe.”*

C018: “*So all the hard ones that we all grapple with*,* they are not there. IVF isn’t there*,* cataract operations isn’t there*,* stuff that would be big volume*,* big money…So the best thing would be to hit some of the really contentious big ones that we know would make a massive difference but will kick off a political storm.”*

The sentiment that the EBI programme fell short of addressing commissioners’ de-adoption policymaking needs was also echoed in relation to List 2 interventions. While the issues related to the pre-existence of policies still arose in relation to List 2, some commissioners did highlight how this second list included more procedures and diagnostic tests for which they did not have pre-existing policies. This did not, however, necessarily lead to local adoption of EBI guidance. A key consideration that arose in relation to List 2 interventions was their *relevance* to commissioning bodies, raising questions around their appropriate audience and levers for implementation.

#### Perceived ‘scope’ of EBI programme interventions indicated limited relevance for commissioners

Respondents indicated that many of the List 2 interventions were outside the usual scope of local commissioning. One informant elaborated on this, by explaining that local NHS commissioning policies tended to be developed if they had potential to influence referrals to secondary (hospital) care for elective procedures, rather than subsidiary interventions that were a smaller component of an episode of secondary care (e.g. use of pre-operative ECG). This underpinned several commissioners’ explanations for why they did not hold pre-existing policies for many of the List 2 interventions:

C004b: “*They [List 2] won’t all be in. If it’s like a diagnostic test*,* for instance*,* probably not*,* whereas the ones where it would be*,* “Right*,* I’m a GP and I’m referring in for a particular procedure*,*” then yes*,* they’re in there*.”

Several commissioners described many of the List 2 interventions as being more akin to guidance for the use of interventions that were part of ‘pathways’ of care that fell within secondary care, thus falling outside of their usual commissioning remit:

C001: “*When the second tranche [List 2] came out and it was more about hospital procedures and diagnostics and stuff*,* that didn’t make - you know*,* sort of very little interest to us. We don’t commission that specifically*,* that sort of pathway*.”

C017: “*I think we were a little disappointed with phase 2 [List 2] if I’m completely honest. There were a lot of things in there that*,* certainly as commissioners*,* we felt we couldn’t do a huge amount with. They’ve got a very much internal pathway in the Trust [hospital]*,* very specific things*.”

As such, commissioners reported passing such EBI guidance on to secondary care providers to decide locally about whether or how they needed to be implemented:

C014b: “*Yeah*,* so there was quite a lot that we didn’t actually take on board and because the providers had been given [by the CCG] all that information [EBI guidance]*,* it was up to them to actually update their information and to look at their own internal policy process and what they’re doing with that.”*

Building on the perception that much of List 2 was outside of commissioners’ scope, several identified that monitoring activity of these interventions would be challenging as often hospitals were reimbursed a fixed amount for a complete episode of care, rather than for individual components of care within that episode, such as those targeted by the EBI programme. Without a way of monitoring activity, commissioners questioned the purpose and value of introducing new commissioning policies for these areas:

C016: “*Yes*,* so I think there were a number of items in List 2 that didn’t set themselves to a commissioning policy…especially with a lot of the things in EBI 2 where they weren’t able to set any targets*,* because it’s not possible to monitor activity. Essentially*,* it’s not possible to monitor a policy. And how…if you develop it*,* what do you then do afterwards?”*

Overall, while commissioners supported the intentions of the EBI programme, there were clear recurring indications that the initiative fell short of addressing their de-adoption policymaking needs, raising questions about the programme’s value. Although not always explicitly discussed, the shortfalls described recurringly linked to limited potential to reduce activity rates.

### Role of local context in shaping implementation of the EBI recommendations

#### Less stringent national policies had little local appeal

Many commissioners viewed the EBI programme as providing a minimum threshold for criteria that needed to be met to access the targeted interventions. The List 1 written guidance allowed discretion for commissioners to impose more stringent criteria for accessing an intervention locally (e.g. mandating a longer duration of conservative management before surgery is considered). Compared to EBI guidance, many reported that their local policies were already more stringent, and therefore could continue to be implemented without adjustment:

C014: “*But we were given the scope [by NHS England]*,* that if our policies were more stringent*,* then we could keep to that. We didn’t have to make them less stringent because obviously that would then be an increase in cost*,* an increase in activity.”*

The perception of EBI policies being less restrictive led some commissioners to indicate that adoption of EBI guidance would have led to an unwelcome increase in activity:

C017: “*We looked [at List 1] and went*,* “Well*,* yeah*,* that’s fine but if we change to that we’re doing more*,* and we don’t really want to be doing more.”*

Concerns about expectations of increasing local activity and financial implications of doing so within a restricted budget were also expressed in relation to List 3 of the EBI programme, which was still being developed at the time of interviews. Some commissioners expressed concerns about the possibility of limited commissioner involvement in future EBI iterations and subsequent increased spending – something that was not felt to be possible in the economic climate. As highlighted by C015, the resource-management function of some local commissioning policies was perceived to clash with proposed national EBI guidance:

C015: “*Some of the List 3 proposals*,* which are currently out for consultation*,* are actually more liberal and looser than current local policy. So they are not going to save resources […]. Some of these EBIs [recommendations] appear to be more liberal than that*,* and that is going to be an issue locally*,* where there might be conflict between what has been made as a decision to manage resources locally*,* where the accountability is for the resources*,* and then EBI comes along and says*,* “Well*,* we are saying you should be using this more than you actually are locally”*.”

#### Complexities of integrating the EBI programme within pre-existing policy-development processes

Implementing EBI recommendations was not a simple process of adopting and abiding by national criteria. Interviews revealed that the prospect of including EBI policies in the local policy portfolio, either as a new addition, or through an amendment to an existing policy - did not exempt them from pre-existing local policy development and ratification processes:

C014b: “*No*,* it [EBI guidance] would follow exactly the same process*,* every policy will follow the same process and pathway through the CCPF [Clinical Commissioning Policy Forum] and all the other things. And everything that we do*,* preparing it*,* getting it ready for review*,* it would follow the same process.”*

Commissioners framed their local policy development/ratification processes as well-established, multi-staged, and often lengthy, processes:

C020: “*We start and look at the evidence base*,* the clinical position*,* and so on. We start from that and then work through the usual process to get involvement*,* engagement. All the various impact analyses of that*,* equality impact analysis*,* quality impact analysis*,* etc. Then come up with a draft*,* which is then sent around for everyone to have a look at and give their views and comments on. Again*,* that will happen*,* and again… Then we’ve got the final draft for approval and adoption.”*

**Fig. 1:**
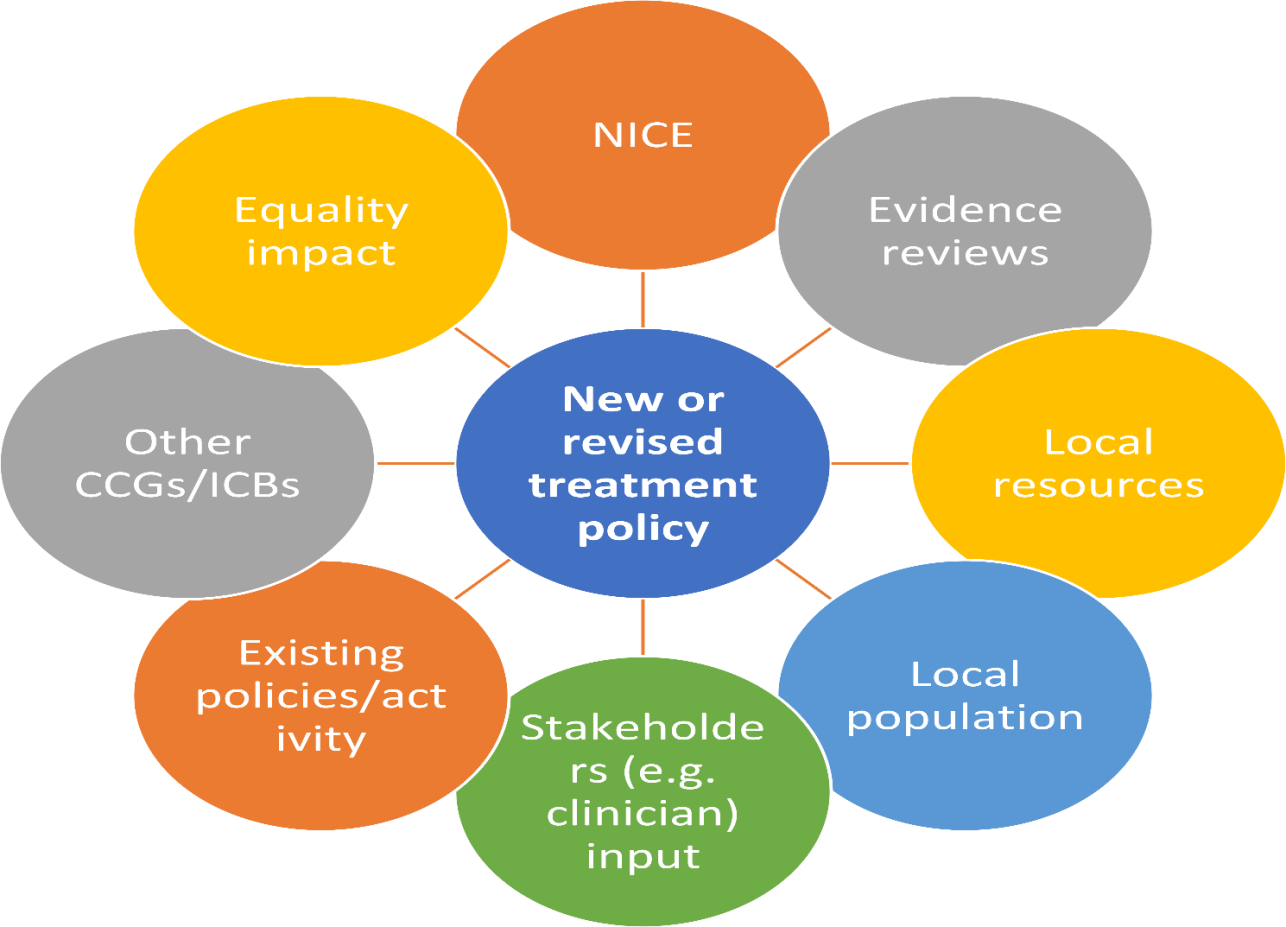
Commissioning policy development process

Although processes were not standardised across commissioning organisations, others described similar components of the process as respondent CO20. The range of factors and considerations commissioners referred to are represented in Fig. 1. As shown, devising commissioning policies comprised a blend of locally bound information (e.g., local activity rates, stakeholder views) and ubiquitous factors (e.g. the evidence-base and (inter)national guidance). As such, local policies were inherently different to the EBI recommendations: the evidence was considered as a part of holistic decisions about where to set thresholds for activity, with local resources and prioritisation in mind.

Passing EBI recommendations through commissioning bodies’ own policy-development processes reportedly highlighted deficiencies in information about how the national recommendations had been developed. This raised challenges for ratifying national EBI recommendations. For example, evidence appraisal was consistently described by all commissioners who discussed their own policy-development processes. This involved reviewing resources that were perceived to be trustworthy, such as NICE guidelines, as well as conducting in-house reviews of existing evidence. While commissioners were confident about the evidence underpinning their locally-developed policies, some expressed scepticism about how evidence had informed national EBI recommendations – especially where the evidence cited did not align with evidence referenced in their pre-existing policy for a given intervention:

C001: “*Because I was involved in it [local policy development] I could see it from start to finish*,* whereas the EBI stuff has been given to us. I don’t know quite how robustly they are all produced. I don’t like things to be produced by consensus*,* because it tends to be the easiest route for everybody concerned. That may not necessarily be the right thing. Whereas when we’ve got hard and fast evidence that can be documented*,* relied upon and used for challenges then I know that I’m on good ground*.”

C016: “*There were a number of cases in which the references provided as part of EBI did not always support the recommendations used within the policy. I think that here*,* quite a bit of it was clinical consensus*,* as opposed to evidence based*,* ignoring the title of the policies*.”

There were exceptions to the above. One commissioner, for example, noted that they had not thought to question the evidence upon which the EBI recommendations were based – referring to their trust in the EBI process:

C018: “*I think*,* because these came out the way they did*,* we kind of trusted… I didn’t check the evidence. So*,* no*,* I must admit I haven’t challenged or tested where*,* what that is. And I am not sure it was ever*,* I mean*,* it was probably communicated but I am not sure I read it*,* if you know what I mean? It was there*,* but I wouldn’t have opened it and gone through it all.”*

The above challenges highlight the difficulties of introducing national policy/recommendations in a context where devolved policymakers have historically had to develop and sustain their own processes. Some interviews also highlighted the time required to work through the above processes, as a reason why local implementation of EBI policies had either not (yet) been possible or straightforward. Commissioners’ accounts of how ‘local consultation’ was a critical aspect of their policy development – particularly with clinicians and patient and public representatives - further highlighted that national policies could not be implemented without substantial local work:

C001: *“Locally we are very*,* I’d not say militant*,* but we are very strong in our clinical views and the need for clinical representation at decision-making arenas*,* especially where clinical pathways and things are discussed*,* because without that clinical input*,* you know*,* it’s left to people who may or may not appreciate some of the nuances in things that are said or not said.”*

Following development of the draft policy/updated policy, commissioners described the need for final ratification by other committees and governance structures prior to implementation. All in all, the process of introducing and amending policies was often described as lengthy and complicated:

C0009: “*So*,* each policy would take 6–12 months to sign off. And there’d be several iterations. And then we’d have to present it to the governing body. And the hospitals. And the council.*”

During the period in which interviews were conducted, many CCGs/ICBs had not yet fully returned to pre-COVID timelines for ratifying policies. In addition, the move from CCGs to ICBs also meant that systems/processes often needed adapting, along with changes to management structures and committee processes. These contextual factors further delayed progress, in relation to the array of decisions and actions that enveloped implementation of the EBI progamme’s national de-adoption recommendations.

## Discussion

This study aimed to investigate the implementation of a de-adoption programme (the EBI programme) within the English NHS, by focusing on key policy-influencers’ (commissioners’) perspectives and experiences of adopting these national recommendations within their local systems. We found that commissioners were supportive of the ethos of using evidence-based national criteria to guide equitable provision of care, but faced a host of barriers in adopting the national recommendations. These challenges revolved around misalignment between national and local bodies in how priorities are met, particularly in the context of restricted budgets, and difficulties assimilating national and local policy development processes.

Since initiation of this study, two independently conducted evaluations of the quantitative impact of the EBI programme concluded that the programme did not appear to facilitate reductions in the 17 List 1 procedures [9, 17]. The qualitative evidence generated through our present study illuminates reasons why the programme appeared to have had little impact on procedure rates. One of the key findings from this study related to the local relevance of the areas identified by the EBI programme for de-adoption, with commissioners describing the List 1 criteria as the “*usual suspects*” for which pre-existing, often more stringent, policies were often already in place. Other international de-adoption programmes, such as the Choosing Wisely Campaign, have been critiqued for similar reasons, whereby the targets of the de-adoption programme have been recognised as “uncontroversial” [4]. In alignment with recommendations from previous research (e.g. the Australian SHARE initiative [22]), the EBI programme was transparent about how it selected its initial list of procedures for de-adoption [23]. As outlined in accompanying guidance for the EBI programme, List 1 procedures were selected due to the anticipation that these were already deemed to be familiar areas that commissioning bodies had already considered for de-adoption. At the time, this was anticipated to facilitate engagement with the programme – or rather, prevent resistance. Future de-adoption efforts may need to consider the tension between wanting to build support and awareness of the campaign (and therefore starting with the ‘usual suspects’) and achieving impact that warrants the effort invested in launching such programmes.

The integration of the EBI programme into the NHS Standard Contract (until 2023/2024) allowed commissioners to refuse payment to a provider if an intervention was not carried out in accordance with EBI guidance. In their scoping review of understanding de-adoption of low-value clinical practices, Niven et al. identified that common mechanisms of facilitating de-adoption processes included restructuring funding associated with the practice that is to be de-adopted, as well as changing policies associated with the procedure [1]. In addition, Elshaug et al. noted the use of partial or full removal of funding to support de-adoption efforts where practice persists despite evidence of ineffectiveness [24]. Our findings, however, highlighted that for financial levers to be applicable, the intervention to be de-adopted needs to be something that can be measured and influenced by those with healthcare purchasing responsibility. Future de-adoption programmes should consider where in the system financial levers are the most applicable. This may be dependent on the health system in question, as well as the audience the policy is directed towards.

Previous de-adoption programmes have highlighted the value policy-makers place on expert involvement and clinical input [3, 25, 26]. We found that consultation with key local stakeholders - specifically, clinicians - was incorporated into commissioners’ overarching perception of the need to account for ‘local requirements’ when implementing the EBI programme. Local clinicians’ acceptance of EBI recommendations was integral to the commissioners’ role in incorporating these national guidelines into local policies. A Delphi study (published in 2014) of factors and processes that facilitate successful de-adoption decisions highlighted the need to engage and involve clinical leaders from an early stage [27], and although there was clinician involvement in formulation of the EBI recommendations (at a national level), this did not necessarily mean that recommendations would be accepted by ‘local’ clinicians. This is important as the understanding and acceptance of evidence and economic evaluation amongst local clinicians and those on the national group may differ. It is also possible that local clinicians have a more powerful voice in local committees compared to the relative power within a national forum. Clinicians’ responses to the EBI recommendations were investigated as part of the OLIVIA study and will be reported in a forthcoming publication.

Flexibility to adapt the List 1 EBI recommendations to fit with local context was perceived to be permitted by commissioners in this study. This may have stemmed from the List 1 guidance [23] which indicated that CCGs had scope to keep or adopt more stringent policies. This highlights a tension with the wider ethos of ensuring criteria are ‘evidence-based’ and equitable. Others have raised similar concerns: research into the provision of varicose vein treatment [28], for instance, has suggested that implementation of the EBI programme was associated with less compliance with evidence-based guidelines (NICE guideline CG168). Future national de-adoption programmes will need to reconcile ‘local context’ (such as available resources and local clinical opinion) with widely accepted priorities of reducing variation. Further research could also helpfully explore commissioners’ views around the trade-offs between variation and local autonomy over healthcare provision.

### Strengths and limitations

This study involved healthcare commissioners from 14 different CCGs/ICBs, meaning the sample was geographically diverse and included a range of professionals involved in the commissioning process, including both managers and clinicians.

The constantly evolving NHS landscape impacted all stages of this study. The transition from CCGs to ICBs meant that commissioners reported their workload as being higher, with many facing harmonisation work to ensure policies aligned across newly formed ICBs. This state of flux meant commissioning policies were constantly evolving, making it sometimes challenging to clearly relate changes in policies to the EBI programme. Conducting interviews over a longer period of time might have revealed different findings, as ICBs became more established. Despite the efforts of the research team, commissioners from some regions were not included in the final sample.

Whilst conducting this research during a period of transition for the NHS could be construed as a limitation, the findings reflect the context of both our research and the implementation of the EBI programme. Any future de-adoption programmes will need to be flexible and amenable to adaptations and ongoing changes in the health system. In addition, the EBI programme itself is evolving, with a change in May 2023 away from the ‘list’ based system and the removal of the statutory obligation to implement the programme from the NHS 2023/2024 Standard Contract [29].

###  Implications

The findings reported here have practical implications for the development of future de-adoption programmes that are intended for meso-level commissioning organisations. In order to be most useful for healthcare commissioners national de-adoption programmes should: (i) consider the resource implications of national guidance, particularly if less stringent than existing local criteria; (ii) focus on interventions within the scope of commissioners where they have the financial (or other) levers to influence practice and the means to monitor progress; and (iii) work with commissioners to develop local implementation processes to minimise duplication and reduce delay.

## Conclusion

This is, to our knowledge, the first investigation of how local healthcare policymakers respond to national de-adoption recommendations. Despite previous research highlighting a desire for more central support in de-adopting healthcare, our study has unearthed the challenges of reconciling national de-adoption recommendations with local policy-development processes and priorities in systems with devolved models of purchasing/providing healthcare. Our findings highlight that de-adoption of embedded healthcare interventions can garner support via the ethos of upholding evidence-based medicine and reducing variation, but implementation of national criteria to guide provision of care is not necessarily a straightforward process – largely due to the fact that de-adoption concerns entrenched (rather than new/innovative) interventions. Future national de-adoption programmes should be prepared to produce guidance and support for addressing resource-related and operational challenges of adopting centralised policies for guiding provision of established interventions, for which local/devolved access arrangements may already exist.

## Supplementary Information

Supplementary Material 1.

Supplementary Material 2.

## Data Availability

The supporting data underpinning this research are ‘controlled data’. Details of how to request access are available via the University of Bristol: https://www.bristol.ac.uk/staff/researchers/data/accessing-research-data/.

## Abbreviations

CCG: Clinical Commissioning Group
EBI: Evidence-based Interventions
ICB: Integrated Care Board
NHS: National health service
NIHR ARC: National Institute for Health and Care Research Applied Research Collaboration
NIHR BRC: National Institute for Health and Care Research Biomedical Research Centre
