# Supplementary material for: Relevance and flexibility are key: exploring healthcare managers’ views and experiences of a de-adoption programme in the English National Health Service
Source: BMC Health Serv Res. 2025 Apr 24;25:590. doi: 10.1186/s12913-025-12700-1 (PMC12020301; doi:10.1186/s12913-025-12700-1)
Supplement: Supplementary file 1 — Supplementary Material 1. [file 12913_2025_12700_MOESM1_ESM.docx]

**Process of generating rankings of CCGs by activity**

1.
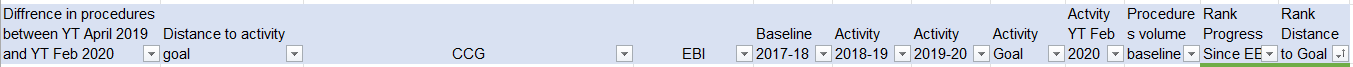
The EBI dashboard (NHS BUSINESS SERVICES AUTHORITY. 2020. Evidence Based Interventions [Online]. Available: https://catalyst.services.nhsbsa.nhs.uk/analytics/saw.dll?Dashboard [Accessed 30th September 2020 2020]) was downloaded, which included CCG activity for each of the List 1 EBI interventions. Figure 1 indicates the data types available.
2. Data on activity were extracted for the three ‘case study’ procedures: tonsillectomy for recurrent tonsillitis, Dupuytren’s contracture release, and arthroscopic shoulder decompression for subacromial pain.
3. CCGs were then ranked according to the progress they had made in reducing procedure numbers. This was done by dividing the CCG’s “Activity 2019-20” by their “Activity YT (year to) Feb 2020”. This produced a figure for the difference in procedures between the year to April 2019 and the year to February 2020. February 2020 was chosen as a cut off in order to mitigate for the impact of COVID -19 on procedure rates.
4. The CCGs that ranked in the ‘top 10’ and the CCGs that ranked in the ‘bottom 10’ in terms of the progress they hade made in reducing procedure rates were identified to be sampled.
