# Supplementary material for: Relevance and flexibility are key: exploring healthcare managers’ views and experiences of a de-adoption programme in the English National Health Service
Source: BMC Health Serv Res. 2025 Apr 24;25:590. doi: 10.1186/s12913-025-12700-1 (PMC12020301; doi:10.1186/s12913-025-12700-1)
Supplement: Supplementary file 2 — Supplementary Material 2. [file 12913_2025_12700_MOESM2_ESM.pdf]

Topic guide: Evaluation of the Evidence-Based Interventions programme

Background: participant's role and organisation

- Could we start by you just telling me a little bit about yourself and your role?
    - Educational background
    - Previous roles
    - How did they become involved in commissioning?
    - What is their role within the commissioning group?
- What geographic area does your work cover?
- I'm aware there have been some changes to CCG structures recently. Have there been any changes to your organisation since April 2019?
- In terms of an integrated care system, has this changed things for you at all?
    - Has the ICS changed how the CCG delivers its functions?
    - Going forward, will there be any changes to where/who devises commissioning policies?

Attitude and understanding of de-adoption and the EBI programme

- When did you first become aware of the EBI programme?
    - How did you first become aware of it? Communications from EBI, local organisations, word of mouth?
- What is your understanding of the EBI programme and what it's setting out to achieve?
- How does this compare with what your CCG/organisation usually does?
    - Was the EBI programme different?
    - If so, how?
    - Did you find it helpful?
- Do you think the EBI programme is needed?
    - Any particular reasons why?
- Do you get a sense of what others within your CCG think about the EBI programme?
    - Other commissioners
    - Clinicians
    - Hospital managers
- In terms of the 4 'do not do' 'category one' procedures, how did the CCG react to these?
    - Are zero payment tariffs in place?
- Have views changed over time (e.g. between List 1 and List 2)?
- What impact or changes has the EBI programme had for your organisation?
    - Policies for accessing procedures?
    - Ways in which policies are implemented?
    - Ways in which care is delivered (e.g., by whom, where)?
    - Ways in which care is paid for? (block funding vs payment by results)

- How will the delivery of the EBI programme proceed in the ICS landscape?
    - Who will champion it?
    - Any significant changes?
- Other than through the EBI programme, have you had any processes locally for sopping or reducing healthcare for whatever reason?
    - Processes for identifying procedures
    - Processes for formulating policy
    - Processes for implementation
    - Processes for evaluation
- How well do you think these processes worked, in practice? (Explore challenges, solutions, positive lessons…).
- How do you think the EBI programme compares to the above?

<u>Case study specific</u>

- We were interested in looking at several of the case study procedures in a bit more detail in this project, as a means of bringing in clinicians' and patients' perspectives. Were any of the EBI procedures from List 1 particularly notable for you?
- How did the EBI criteria compare to any policies you had before for [procedure]
    - Any sense for why the EBI criteria were different?
    - Were any actions taken to accommodate the EBI criteria?
- Have your existing policies had to change significantly or were they already in line with the EBI programme – in particular for:
    - Tonsillectomy
    - Dupuytren's contracture release
    - Arthroscopic sub-acromial decompression
- Did you engage with other stakeholders following the EBI recommendations for [procedure]?
    - Clinicians, hospital managers, patients, public, others?
    - What did this entail?
    - How did it go? Any difficulties faced? How were these resolved?
- What impact do you think these changes have had, in terms of activity for [procedure]?
    - Why do you think this is the case?
- Have there been any implications or consequences of these changes for different groups?
    - Patients?
    - Clinicians?
    - Hospital managers?
    - Commissioners?

<u>Impact of COVID-19</u>

- How has the COVID-19 pandemic affected the EBI programme and its delivery?
    - Are there any lasting implications?
- How did the pandemic impact the provision of elective procedures in your CCG?
- What are the implications of this for the future?

Future of EBI

- To end the interview, it would be helpful to hear your thoughts on what's working well with EBI, and what you think could be changed, if the programme were to carry on.
    - Can we start with what, if anything, is going well?
    - And how about things that could change?

Thank for taking part, ask if they have any additional questions or anything they would like to add. Remind will send consent form – but they don't need to take any action with it.
